# Evaluation of formaldehyde when complete feed and soybean meal were inoculated with porcine epidemic diarrhea virus, porcine reproductive and respiratory syndrome virus, and Seneca Valley virus 1

**DOI:** 10.1093/tas/txae121

**Published:** 2024-08-23

**Authors:** Olivia L Harrison, Jianfa Bai, Martee Larson, Roman M Pograninchniy, Francisco Domingues, Nicole Holcombe, Othmar Lopez, Cassandra K Jones

**Affiliations:** Department of Animal Sciences and Industry, Kansas State University, Manhattan, KS 66056, USA; Department of Diagnostic Medicine/Pathobiology, Kansas State University, Manhattan, KS 66056, USA; Department of Diagnostic Medicine/Pathobiology, Kansas State University, Manhattan, KS 66056, USA; Department of Diagnostic Medicine/Pathobiology, Kansas State University, Manhattan, KS 66056, USA; Anitox Corporation, Lawrenceville, GA 30043, USA; Anitox Corporation, Lawrenceville, GA 30043, USA; Anitox Corporation, Lawrenceville, GA 30043, USA; Department of Animal Sciences and Industry, Kansas State University, Manhattan, KS 66056, USA

**Keywords:** feed, formaldehyde, soybean meal, viruses

## Abstract

Formaldehyde has been found to decrease virus concentrations in feed and ingredient matrices. Continued research is needed to identify the appropriate inclusion levels and application time for different viruses in these matrices. The objective was to evaluate different inclusion levels of formaldehyde when applied either pre- or postinoculation of porcine epidemic diarrhea virus (**PEDV**), type 2 porcine reproductive and respiratory syndrome virus (**PRRSV**), and Seneca Valley virus 1 (**SVV1**) to complete feed or soybean meal. The experiment was designed in a 2 × 2 factorial with a formaldehyde-based product (Termin-8, Anitox Corporation, Lawrenceville, GA) applied either before virus inoculation (preinoculation) or after inoculation (postinoculation) at either a 2 or 3 kg/MT. On day 0, samples of the respective matrices were weighed in 50 g aliquots and added to 500 mL bottles. Formaldehyde was applied to the preinoculation samples at the respective inclusion levels and 50 µL of each virus were added to the postinoculation samples. All bottles were shaken and allowed to sit at room temperature for 24 h. On day 1, virus was added to the preinoculation samples and formaldehyde was added to the postinoculation bottles. Half of the samples were immediately processed (0 h) and the other half were incubated at room temperature for an additional 24 h. Samples were processed and aliquots were analyzed via triplex PCR. An application time × inclusion level interaction was observed for PEDV at 0 h and SVV1 and PEDV at 24 h in complete feed, where less viral RNA (*P* < 0.05) was detected in the postinoculation samples at either inclusion level as compared to the positive controls. In soybean meal, the same interaction was observed in PEDV and PRRSV at 0 h and SVV1 and PEDV at 24 h with less detectable RNA observed (*P* < 0.05) in the postinoculation samples regardless of inclusion level than the preinoculation counterparts and the controls. Overall, an application time effect was noticed in each matrix where less RNA was detected in the postinoculation samples at 0 h (*P* < 0.05) compared to the preinoculation samples and the control, and at 24 h, both the pre- and postinoculation samples had less detectable RNA (*P* < 0.05) than the control. Overall, formaldehyde can reduce detectable RNA immediately in contaminated complete feed and soybean meal, with greater decreases observed as mitigant contact time increases.

## Teaser

The use of formaldehyde can reduce the quantity of porcine epidemic diarrhea virus, porcine reproductive and respiratory syndrome virus, and Seneca Valley virus 1 RNA in complete feed and soybean meal immediately after application and after a 24-h incubation.

## Graphical Abstract

**Graphical Abstract:**
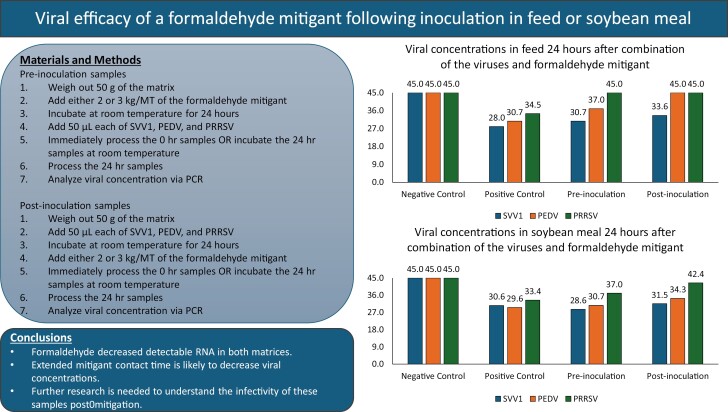

## Introduction

Since the outbreak of porcine epidemic diarrhea virus (PEDV) in 2013, there has been increased research evaluating the mitigation of feed to reduce viral presence in feed. Formaldehyde is a chemical mitigant which is hypothesized to bind to viral protein and genetic material which limits the virus’ ability to replicate inside the host cell (McDonnell and Russell, 1999). Inclusion of formaldehyde has decreased PEDV, type 2 porcine reproductive and respiratory syndrome virus (PRRSV), and Seneca Valley virus 1 (SVV1) concentrations in complete feed and reduced the risk of viral infection compared to untreated feed (Dee et al., 2021). Feed ingredients may also harbor viral pathogens prior to entering the feed mill. Specifically, SVV1 and African swine fever virus (**ASFV**) have remained viable for extended durations in unmitigated soybean-based ingredients (soybean meal and soy oilcake) as well as complete feed (Dee et al., 2018).

Due to the risk of viral pathogens entering the feed supply chain, it is imperative to understand the best practices when including formaldehyde in the feed. These practices include both the time of mitigant application and the inclusion level needed for viral inactivation. Lerner et al. (2020) found no differences when comparing the timing of chemical mitigant application, either prior to or after PEDV inoculation, on the detection of genetic material, but this occurrence has not been evaluated in ingredient matrices or using other viruses. Formaldehyde included at 0.35% (3.5 kg/MT) reduced ASFV concentrations below detectable limits in cell culture alone in as little as 30 min (Niederwerder et al., 2021); however, the presence of feed is likely to increase the contact time needed for viral inactivation. Therefore, the objective of this study was to evaluate the effect of formaldehyde inclusion at varying inclusion levels when SVV1, PEDV, and PRRSV were included either prior to or after mitigant application in both complete feed and soybean meal at 2 different time points.

## Materials and Methods

### Inoculum Information

An equal volume of SVV1 (GenBank: KX7780101.1), PEDV CO-isolate (GenBank KF272920), and PRRSV 1-7-4 (GenBank: PP239061) were used for feed inoculation. The original stock contained 1 × 10^8^ 50% median tissue culture infectious dose/mL (10^8^ TCID_50_/mL) SVV1, 1 × 10^7^ TCID_50_/mL PEDV, and 1 × 10^8^ TCID_50_/mL PRRSV. Viruses were individually packaged into 25 mL aliquots, shipped from South Dakota State University to Kansas State University on dry ice, and stored at −80 °C. Prior to the start of the experiment, a 25-mL aliquot of each virus was thawed and divided into four 900 µL aliquots to reduce the freezing and thawing of the stock virus. On the day of inoculation, a 900-µL aliquot of each virus was removed from storage and allowed to thaw at room temperature.

### Complete Feed and Soybean Meal Information

A corn–soybean meal gestation diet was manufactured in meal form at O.H. Kruse Feed Technology Innovation Center (Manhattan, KS). Soybean meal was also sourced from the O.H. Kruse Feed Technology Innovation Center from the bulk ingredient storage. Prior to inoculation, 50 g of each matrix was weighed and placed in 500 mL high-density polyurethane bottles.

### Inoculation and Formaldehyde Application

The experiment was designed as a 2 × 2 factorial at 2 different time points, where mitigant application time (pre- or postinoculation) by inclusion level (2 or 3 kg/MT) was evaluated either immediately after both the mitigant and virus were combined (0 h) or after 24 h. There were 3 replicates for each application time × inclusion level sample at each time point.

At the start of the experiment (day 0), bottles with 50 g of complete feed were separated by the time of inoculation (pre- or postinoculation). Formaldehyde (Termin-8, Anitox Corporation) was applied to the preinoculation samples at either 2 or 3 kg/MT inclusion levels; bottles were shaken for approximately 10 s to evenly distribute the mitigant. For the postinoculation samples, 50 µL of each virus was added to the bottle and shaken for approximately 10 s to evenly distribute the virus in the feed. Both pre- and postinoculation bottles were incubated at room temperature for 24 h. On day 1, 50 µL of each virus was added to the preinoculation samples and either 2 or 3 kg/MT of formaldehyde was added to the postinoculation samples, all bottles were shaken for even product or virus distribution. Following the inclusion of either the virus for the preinoculation samples or the mitigant for the postinoculation samples, hour 0 samples were collected. The remaining bottles were stored for an additional 24 h at room temperature.

The negative control was not inoculated or mitigated. Two positive controls were included, one inoculated on day 0 as the postinoculation control and the other on day 1 as the preinoculation control. The sample inoculation and mitigant application process was repeated with soybean meal.

### Sample Processing

Following the respective incubation time for the 0 or 24 h samples, 200 mL of phosphate-buffered solution was added to each feed/ingredient sample to create a 20% suspension. Feed/ingredient samples were shaken for approximately 10 s and stored at 4 °C overnight. The next day, 25 mL of the supernatant from the feed samples were transferred to fresh 50 mL conical tubes. The supernatant was centrifuged at 4,000 × *g* for 10 min at 8 °C. Two 300 µL aliquots were retained for PCR analysis and 20 mL was transferred to a fresh 50 mL conical tube for a bioassay.

### Quantitative Viral Analysis

Samples were analyzed for detection and quantification of SVV1, PEDV, and PRRSV using a quantitative reverse transcription real-time polymerase chain reaction (qRT-PCR) assay at the Kansas State University Veterinary Diagnostic Laboratory. First, 50 µL of supernatant was placed in a deep well plate and RNA was extracted using a Kingfisher Flex magnetic particle processor (Fisher Scientific, Pittsburgh, PA) and a MagMAX-96 Viral Isolation Kit (Life Technologies, Grand Island, NY). The final elution volume was reduced to 60 µL, and extracted RNA was stored at −80 °C until analyzed for SVV1, PEDV, or PRRSV using the qRT-PCR triplex assay with a maximum amplification cycle of 45. Results were reported as the number of samples considered positive and the cycle threshold (Ct) below 45 at which either SVV1, PEDV, or PRRSV RNA was detected.

### Statistical Analysis

The application time × inclusion level interaction and subsequent main effects were analyzed as a completely randomized design using the GLIMMIX procedure of SAS (v. 9.4, SAS Institute Inc., Cary, NC) with sample bottle as the experimental unit. Fixed effects included the mitigant application time (pre- or postinoculation), the mitigant application level (2 or 3 kg/MT), and their associated interactions. Data were separated and individually analyzed based on the matrix (complete feed or soybean meal), time of sampling (0 or 24 h), and virus (SVV1, PEDV, and PRRSV). The Ct of each sample was used to estimate the quantity of detectable viral RNA. If no viral RNA was detected, samples were assigned a value of 45. Contrast statements were utilized to compare the 0 and 24 h samples for each mitigant application time × inclusion level comparison. A Kenward–Roger denominator degree of freedom adjustment was used, as well as a Tukey–Kramer multiple comparison adjustment. Results were considered significant at *P* ≤ 0.05.

## Results

### Complete Feed

As expected, no viral RNA was detected in the negative control samples. At 0 h post-application of both the viruses and formaldehyde, there is a formaldehyde application time × inclusion level interaction for PEDV where less detectable RNA (*P* < 0.05) is present when formaldehyde is applied postinoculation at either 2 or 3 kg/MT (Table 1). An interaction was not observed (*P* > 0.05) for either SVV1 or PRRSV, but for both viruses less viral RNA was detected (*P* < 0.05) when formaldehyde was applied postinoculation than when applied prior to viral inoculation (Table 2) regardless of inclusion level.

**Table 1.** Effect of application time and formaldehyde inclusion level on the presence and relative quantification of SVV1, PEDV, and PRRSV RNA in complete feed^1^^,^^2^^,^^3^^,^^4^

	Negative control^2^	Preinoculation			Postinoculation				
		Positive control	2 kg/MT	3 kg/MT	Positive control	2 kg/MT	3 kg/MT	SEM	*P*
0 h									
SVV1	45.0 (0/3)	25.1 (3/3)	25.6 (3/3)	25.9 (3/3)	26.2 (3/3)	28.5 (3/3)	29.3 (3/3)	0.74	0.098
PEDV	45.0 (0/3)	30.1^b^ (3/3)	30.4^b^ (3/3)	30.6^b^ (3/3)	31.1^b^ (3/3)	34.4^a^ (3/3)	36.1^a^ (3/3)	0.83	0.007
PRRSV	45.0 (0/3)	35.5 (3/3)	36.6 (3/3)	38.9 (2/3)	35.1 (3/3)	42.7 (1/3)	42.4 (1/3)	2.70	0.265
24 h									
SVV1	45.0 (0/3)	27.8^d^ (3/3)	30.0^c^ (3/3)	31.4^bc^ (3/3)	28.3^d^ (3/3)	32.6^b^ (3/3)	34.5^a^ (3/3)	0.50	0.007
PEDV	45.0 (0/3)	30.5^c^ (3/3)	34.4^bc^ (3/3)	39.6^ab^ (2/3)	30.9^c^ (3/3)	45.0^a^ (0/3)	45.0^a^ (0/3)	1.64	0.003
PRRSV	45.0 (0/3)	34.2 (3/3)	45.0 (0/3)	45.0 (0/3)	34.9 (3/3)	45.0 (0/3)	45.0 (0/3)	0.35	0.366

^1^Formaldehyde was applied to the complete feed either prior to inoculation (preinoculation) or after inoculation (postinoculation). Formaldehyde was not applied to the positive controls, but due to the different times of inoculation, 2 positive controls are included in the analysis. A cycle threshold (Ct) value of 45.0 is considered negative with no detectable viral RNA.

^2^Negative controls were not included in the statistical analysis.

^3^Presence is determined by the total number of samples that were PCR positive over the total number of replicates (*n* = 3).

^4^Formaldehyde (Termin-8, Anitox Corporation).

^a,b,c,d^Means with differing superscripts within row differ significantly, *P *< 0.05.

**Table 2.** Effect of application time of formaldehyde on the presence and relative quantification of SVV1, PEDV, and PRRSV RNA in complete feed^1^^,^^2^^,^^3^^,^^4^

	Negative control	Positive control^5^	Preinoculation	Postinoculation	SEM	*P*
0 h						
SVV1	45.0 (0/3)	25.6^b^ (6/6)	25.8^b^ (6/6)	28.9^a^ (6/6)	0.53	<0.0001
PEDV	45.0 (0/3)	30.6^b^ (6/6)	30.5^b^ (6/6)	35.3^a^ (6/6)	0.64	<0.0001
PRRSV	45.0 (0/3)	35.3^b^ (6/6)	37.8^b^ (5/6)	42.8^a^ (2/6)	1.76	0.003
24 h						
SVV1	45.0 (0/3)	28.0^c^ (6/6)	30.7^b^ (6/6)	33.6^a^ (6/6)	0.53	<0.0001
PEDV	45.0 (0/3)	30.7^c^ (6/6)	37.0^b^ (5/6)	45.0^a^ (0/6)	1.41	<0.0001
PRRSV	45.0 (0/3)	34.5^b^ (6/6)	45.0^a^ (0/6)	45.0^a^ (0/6)	0.25	<0.0001

^1^Formaldehyde was applied to the complete feed either prior to inoculation (preinoculation) or after inoculation (postinoculation). The means presented are averaged across the inclusion level (2 or 3 kg/MT). A cycle threshold (Ct) value of 45.0 is considered negative with no detectable viral RNA.

^2^Negative controls were not included in the statistical analysis.

^3^Presence is determined by the total number of samples that were PCR positive over the total number of replicates (*n* = 3).

^4^Formaldehyde (Termin-8, Anitox Corporation).

^5^Positive controls are the average of both the prevention and mitigation positive samples.

^a,b,c^Means with differing superscripts within row differ significantly, *P *< 0.05.

After 24 h, an application time × inclusion level interaction was observed for both PEDV and SVV1 (Table 1). Similar to 0 h, there was less detectable PEDV RNA (*P* < 0.05) postinoculation at either inclusion level as compared to formaldehyde application preinoculation. For SVV1, applying formaldehyde at 3 kg/MT postinoculation resulted in less viral RNA (*P* < 0.05) than at 2 kg/MT or at either preinoculation inclusion level. While a PRRSV interaction was not observed, the inclusion of formaldehyde, regardless of the time of application (Table 2) or the inclusion level (Table 3), reduced detectable viral RNA (*P* < 0.05) beyond detectable limits compared to the control. Overall, viral RNA decreased between the 0- and 24-h sampling times (*P* < 0.05; Table 4).

**Table 3.** Effect of formaldehyde inclusion level on the relative quantification of SVV1, PEDV, and PRRSV RNA in complete feed^1^^,^^2^^,^^3^

	Negative control	Positive control	2 kg/MT inclusion	3 kg/MT inclusion	SEM	*P*
0 h						
SVV1	45.0 (0/3)	25.6 (6/6)	27.0 (6/6)	27.6 (6/6)	0.96	0.135
PEDV	45.0 (0/3)	30.6 (6/6)	32.4 (6/6)	33.4 (6/6)	1.36	0.161
PRRSV	45.0 (0/3)	35.4 (6/6)	39.7 (4/6)	40.6 (3/6)	2.14	0.057
24 h						
SVV1	45.0 (0/3)	28.0^b^ (6/6)	31.3^a^ (6/6)	32.9^a^ (6/6)	0.81	<0.0001
PEDV	45.0 (0/3)	30.7^b^ (6/6)	39.7^a^ (3/6)	42.3^a^ (2/6)	2.41	0.0006
PRRSV	45.0 (0/3)	34.5^b^ (6/6)	45.0^a^ (0/6)	45.0^a^ (0/6)	0.25	<0.0001

^1^Formaldehyde was applied at either 2 or 3 kg/MT to complete feed. The means presented are averaged across the time of application (pre- or postinoculation). A cycle threshold (Ct) value of 45.0 is considered negative with no detectable viral RNA.

^2^Negative controls were not included in the statistical analysis.

^3^Formaldehyde (Termin-8, Anitox Corporation).

^a,b^Means with differing superscripts within row differ significantly, *P *< 0.05.

**Table 4.** The effect of time when feed samples were inoculated with SVV1, PEDV, and PRRSV for the quantity of detectable RNA (Ct) following application of formaldehyde incubated for either 1 h or 24 h^1^^,^^2^

	SVV1				PEDV				PRRSV			
	0 h	24 h	SEM	*P*	0 h	24 h	SEM	*P*	0 h	24 h	SEM	*P*
Postinoculation												
Positive	26.2	28.3	0.97	0.097	31.1	30.9	0.76	0.834	35.1	34.9	0.64	0.682
2 kg/MT	28.5^b^	32.6^a^	0.61	<0.0001	34.4^b^	45.0^a^	0.61	<0.0001	42.7	45.0	2.25	0.374
3 kg/MT	29.3^b^	34.5^a^	0.59	<0.0001	36.1^b^	45.0^a^	0.81	0.0004	42.4	45.0	2.60	0.374
Preinoculation												
Positive	25.1^b^	27.8^a^	0.66	0.016	30.1	30.5	0.42	0.391	35.5	34.2	0.77	0.159
2 kg/MT	25.6^b^	30.0^a^	0.40	0.0004	30.4^b^	34.4^a^	0.94	0.013	36.6^b^	45.0^a^	0.14	<0.0001
3 kg/MT	25.9^b^	31.4^a^	0.36	0.0001	30.6^b^	39.6^a^	2.73	0.030	38.9	45.0	3.06	0.116

^1^Complete feed was tri-inoculated with SVV1, PEDV, and PRRSV. Formaldehyde was applied at either 2 or 3 kg/MT postinoculation (formaldehyde applied after viral inoculation) or preinoculation (formaldehyde applied before viral inoculation).

^2^Termin-8, Anitox Corporation.

^a,b^Means with differing superscripts within row differ significantly for each individual virus, *P *< 0.05.

### Soybean Meal

Viral RNA was not detected in the negative control samples. Immediately after the viruses and the formaldehyde were combined (0 h), there was less detectable PEDV and PRRSV RNA (*P* < 0.05) when formaldehyde was applied postinoculation at either inclusion level compared to the positive controls or the preinoculation samples (Table 5). An application time × inclusion level was not observed for SVV1 (*P* > 0.05), but less viral RNA was detected (*P* < 0.05) when formaldehyde was applied postinoculation as compared to both the positive control and the preinoculation application time (Table 6). The inclusion of formaldehyde at either level decreased the quantity of detectable SVV1 RNA (*P* < 0.05) as compared to the positive controls (Table 7).

**Table 5.** Effect of application time and formaldehyde inclusion level on the presence and relative quantification of SVV1, PEDV, and PRRSV RNA in soybean meal^1^^,^^2^^,^^3^^,^^4^

	Negative control^2^	Preinoculation			Postinoculation				
		Positive control	2 kg/MT	3 kg/MT	Positive control	2 kg/MT	3 kg/MT	SEM	*P*
0 h									
SVV1	45.0 (0/3)	23.4 (3/3)	24.7 (3/3)	24.6 (3/3)	23.4 (3/3)	27.3 (3/3)	27.4 (3/3)	0.89	0.086
PEDV	45.0 (0/3)	25.7^b^ (3/3)	26.3^b^ (3/3)	26.2^b^ (3/3)	27.0^b^ (3/3)	29.9^a^ (3/3)	31.1^a^ (3/3)	0.51	0.001
PRRSV	45.0 (0/3)	30.0^b^ (3/3)	31.2^b^ (3/3)	31.2^b^ (3/3)	30.6^b^ (3/3)	34.4^a^ (3/3)	36.3^a^ (3/3)	0.58	0.0006
24 h									
SVV1	45.0 (0/3)	30.9^ab^ (3/3)	28.3^b^ (3/3)	28.9^b^ (3/3)	30.3^ab^ (3/3)	30.5^ab^ (3/3)	32.4^a^ (3/3)	0.90	0.020
PEDV	45.0 (0/3)	29.6^c^ (3/3)	30.2^bc^ (3/3)	31.3^bc^ (3/3)	29.6^c^ (3/3)	33.1^ab^ (3/3)	35.5^a^ (3/3)	0.87	0.016
PRRSV	45.0 (0/3)	33.5 (3/3)	34.8 (3/3)	39.1 (2/3)	33.4 (3/3)	42.3 (1/3)	42.5 (1/3)	2.76	0.188

^1^Formaldehyde was applied to soybean meal either prior to inoculation (preinoculation) or after inoculation (postinoculation). Formaldehyde was not applied to the positive controls, but due to the different times of inoculation, 2 positive controls are included in the analysis. A cycle threshold (Ct) value of 45.0 is considered negative with no detectable viral RNA.

^2^Negative controls were not included in the statistical analysis.

^3^Presence is determined by the total number of samples that were PCR positive over the total number of replicates (*n* = 3).

^4^Formaldehyde (Termin-8, Anitox Corporation).

^a,b,c,d^Means with differing superscripts within row differ significantly, *P *< 0.05.

**Table 6.** Effect of application time of formaldehyde on the presence and relative quantification of SVV1, PEDV, and PRRSV RNA in soybean meal^1^^,^^2^^,^^3^^,^^4^

	Negative control	Positive control^5^	Preinoculation	Postinoculation	SEM	*P*
0 h						
SVV1	45.0 (0/3)	23.4^b^ (6/6)	24.6^b^ (6/6)	27.3^a^ (6/6)	0.57	<0.0001
PEDV	45.0 (0/3)	26.3^b^ (6/6)	26.3^b^ (6/6)	30.5^a^ (6/6)	0.45	<0.0001
PRRSV	45.0 (0/3)	30.3^b^ (6/6)	31.2^b^ (6/6)	35.4^a^ (6/6)	0.52	<0.0001
24 h						
SVV1	45.0 (0/3)	30.6^a^ (6/6)	28.6^b^ (6/6)	31.5^a^ (6/6)	0.68	0.003
PEDV	45.0 (0/3)	29.6^b^ (6/6)	30.7^b^ (6/6)	34.3^a^ (6/6)	0.52	<0.0001
PRRSV	45.0 (0/3)	33.4^b^ (6/6)	37.0^b^ (5/6)	42.4^a^ (2/6)	1.92	0.001

^1^Formaldehyde was applied to soybean meal either prior to inoculation (preinoculation) or after inoculation (postinoculation). The means presented are averaged across the inclusion level (2 or 3 kg/MT). A cycle threshold (Ct) value of 45.0 is considered negative with no detectable viral RNA.

^2^Negative controls were not included in the statistical analysis.

^3^Presence is determined by the total number of samples that were PCR positive over the total number of replicates (*n* = 3).

^4^Formaldehyde (Termin-8, Anitox Corporation).

^5^Positive controls are the average of both the prevention and mitigation positive samples.

^a,b^Means with differing superscripts within row differ significantly, *P *< 0.05.

**Table 7.** Effect of formaldehyde inclusion level on the relative quantification of SVV1, PEDV, and PRRSV RNA in soybean meal^1^^,^^2^^,^^3^

	Negative control	Positive control	2 kg/MT inclusion	3 kg/MT inclusion	SEM	*P*
0 h						
SVV1	45.0 (0/3)	23.4^b^ (6/6)	26.0^a^ (6/6)	26.0^a^ (6/6)	0.90	0.014
PEDV	45.0 (0/3)	26.3 (6/6)	28.1 (6/6)	28.7 (6/6)	1.18	0.152
PRRSV	45.0 (0/3)	30.3^b^ (6/6)	32.8^ab^ (6/6)	33.7^a^ (6/6)	1.17	0.026
24 h						
SVV1	45.0 (0/3)	30.6 (6/6)	29.4 (6/6)	30.6 (6/6)	0.95	0.384
PEDV	45.0 (0/3)	29.6^b^ (6/6)	31.6^ab^ (6/6)	33.4^a^ (6/6)	1.08	0.011
PRRSV	45.0 (0/3)	33.4^b^ (6/6)	38.5^ab^ (4/6)	40.8^a^ (3/6)	2.30	0.017

^1^Formaldehyde was applied at either 2 or 3 kg/MT to soybean meal. The means presented are averaged across the time of application (pre- or postinoculation). A cycle threshold (Ct) value of 45.0 is considered negative with no detectable viral RNA.

^2^Negative controls were not included in the statistical analysis.

^3^Formaldehyde (Termin-8, Anitox Corporation).

^a,b^Means with differing superscripts within row differ significantly, *P *< 0.05.

At 24 h post-application of both the viruses and formaldehyde, an interaction was observed for both PEDV and SVV1, where the inclusion of 3 kg/MT formaldehyde postinoculation reduced the quantity of detectable RNA (*P* < 0.05) as compared to the controls, with other application times and inclusion levels being intermediate (Table 5). Less PRRSV RNA was detected when formaldehyde was applied postinoculation as compared to preinoculation (*P* < 0.05; Table 6) and the inclusion of 3 kg/MT resulted in less detectable RNA (*P* < 0.05) as compared to the untreated, positive control (Table 7). Similar to complete feed, less viral RNA was detected at 24 h as compared to 0 h (Table 8).

**Table 8.** The effect of time when soybean meal samples were inoculated with SVV1, PEDV, and PRRSV for the quantity of detectable RNA (Ct) following application of formaldehyde for either 1 h or 24 h^1^^,^^2^

	SVV1				PEDV				PRRSV			
	0 h	24 h	SEM	*P*	0 h	24 h	SEM	*P*	0 h	24 h	SEM	*P*
Postinoculation												
Positive	23.4^b^	30.3^a^	0.77	0.001	27.0^b^	29.6^a^	0.36	0.002	30.6^b^	33.4^a^	0.64	0.013
2 kg/MT	27.3^b^	30.5^a^	1.11	0.045	29.9	33.1	1.14	0.052	34.4^b^	42.3^a^	2.82	0.050
3 kg/MT	27.4^b^	32.4^a^	0.91	0.005	31.1^b^	35.5^a^	1.06	0.015	36.3	42.5	2.52	0.069
Preinoculation												
Positive	23.4^b^	30.9^a^	1.35	0.005	25.7^b^	29.6^a^	0.55	0.002	30.0^b^	33.5^a^	0.48	0.002
2 kg/MT	24.7^b^	28.3^a^	0.44	0.001	26.3^b^	30.2^a^	0.32	0.0003	31.2^b^	34.8^a^	0.50	0.002
3 kg/MT	24.6^b^	28.9^a^	0.39	0.0004	26.3^b^	31.3^a^	0.20	<0.0001	31.2	39.1	2.93	0.054

^1^Soybean meal was tri-inoculated with SVV1, PEDV, and PRRSV. Formaldehyde was applied at either 2 or 3 kg/MT postinoculation (formaldehyde applied after viral inoculation) or preinoculation (formaldehyde applied before viral inoculation).

^2^Termin-8, Anitox Corporation.

^a,b^Means with differing superscripts within row differ significantly for each individual virus, *P *< 0.05.

## Discussion

To the authors’ knowledge, this is the first experiment to evaluate the effect of both the formaldehyde inclusion level and the time of product application. Application of formaldehyde postinoculation immediately reduced the quantity of PEDV RNA in feed and PEDV and PRRSV RNA in soybean meal. The effect of product application time has only been studied by Lerner et al. (2020), in which there was no difference between pre- and postinoculation samples. It is unclear why the preinoculation samples did not have a similar immediate effect on the quantity of detectable RNA in either matrix.

As the contact time increased to 24 h, greater reductions in viral concentrations were observed, with both pre- and postinoculation feed samples having a larger reduction in the quantity of detectable SVV1 and PEDV RNA as compared to the positive controls. Cochrane et al. (2020) observed a similar effect where the quantity of PEDV RNA did not differ between the positive control and formaldehyde-treated feed on day 0, but following 24 h contact time, the formaldehyde-treated feed had less detectable PEDV RNA. However, in soybean meal, less SVV1 RNA was detected in the 3 kg/MT postinoculation samples as compared to the preinoculation samples at either inclusion level. Soybean meal has often been demonstrated to sustain viruses longer than other matrices like feed (Dee et al., 2015; Caserta et al., 2022; Niederwerder et al., 2022), which may explain the less pronounced effect of formaldehyde as compared to the mitigated complete feed.

Although statistical differences were not observed, the use of formaldehyde reduced PRRSV concentrations beyond detectable means in feed at 0 and 24 h and soybean meal at 24 h. Similarly, multiple mitigated feed samples at 24 h contained no detectable PEDV RNA. Dee et al. (2015) was not able to detect PEDV RNA 7 d after inoculation in formaldehyde-treated complete feed held at freezing temperatures (−18 to −9 °C). As the samples in this study were held at room temperature (approximately 25 °C), the time needed for viral decay is likely to decrease (Mil-Homens et al., 2024) compared to Dee et al. (2015).

Niederwerder et al. (2021) observed decreased detectable ASFV DNA when feed matrices were mitigated with 3.3 kg/MT (0.35% inclusion) of formaldehyde. This inclusion level of formaldehyde also reduced the risk of swine infection via a swine bioassay. In this study, the different inclusion levels had a variable effect on viral concentrations where the mitigated feed at 24 h had less detectable RNA than the positive control at either inclusion level. In soybean meal, however, the highest inclusion level had the greatest difference in viral concentrations. Both inclusion levels in this study decreased the quantity of detectable viral RNA, but the higher inclusion levels (3 kg/MT) had a more consistent effect in both feed and soybean meal.

## Limitations

As this study specifically evaluated the presence of viral RNA in complete feed and soybean meal, the ability of these treated ingredients to cause infection remains unknown. In multiple studies, the presence of viral RNA in treated matrices was unable to cause infection and thus reduced the risk of viral transmission (Dee et al., 2015, 2021; Niederwerder et al., 2021). The use of bioassays or the emerging viable PCR assay (Palowski et al., 2022) are options which may better elucidate the overall effectiveness of formaldehyde in feed and ingredient matrices.

## Conclusions

The use of formaldehyde was able to decrease the quantity of viral RNA in both complete feed and soybean meal. Formaldehyde applied after inoculation immediately reduces more viral RNA than when applied prior to inoculation, but the magnitude of difference lessens after 24 h. Further research is needed to understand the infectivity of these samples and whether greater differences are noticed between the mitigated and unmitigated samples with longer incubation times.
